# Efficacy of Bovine Hydroxyapatite and Collagen Along With Platelet-Rich Fibrin as a Scaffold and Human Chorion as a Membrane for Ridge Preservation: A Case-Control Study

**DOI:** 10.7759/cureus.21362

**Published:** 2022-01-18

**Authors:** Tanuja B, Krishna Mohan Kondareddy, Ramesh A, Rajesh N, Siva Rami Reddy E, Ravi Prakash

**Affiliations:** 1 Department of Periodontics, MNR Dental College and Hospital, Hyderabad, IND; 2 Department of Periodontics, G. Pulla Reddy Dental College and Hospital, Kurnool, IND; 3 Department of Medicine, MNRH Medical College, Hyderabad, IND; 4 Department of Oral Pathology, G. Pulla Reddy Dental College and Hospital, Kurnool, IND

**Keywords:** g - graft, bovine hydroxyapatite, prf, chorion membrane, ridge preservation

## Abstract

Aim: The present study aims to determine the efficacy of bovine hydroxyapatite and collagen (G-graft) mixed with platelet-rich fibrin (PRF) used as a scaffold and chorion membrane as a barrier in post-extraction sockets with extraction sites alone.

Methods and material: Thirty individuals were randomly assigned into two groups. In the control group, after debridement of the extracted tooth socket, no additional treatment was done. In the test group, after debridement of the extracted tooth socket, the sockets were filled with bovine hydroxyapatite and collagen (G-graft) mixed with PRF. They were covered by a chorion membrane, and a non-absorbable suture material was used to secure the membrane in place. Clinical parameters assessed were plaque index, gingival index, buccolingual width, buccal bone plate height, and lingual bone plate height at baseline and at six months.

Results: Clinically, there was a more significant reduction in the buccolingual width of the control group than the test group after six months. A statistically significant difference between the two groups for vertical ridge height at the mesial and distal socket sites was observed. No statistically significant difference in buccal and lingual bone plate height was observed between the two groups (p>0.05).

Conclusions: Both groups showed a significant reduction in the Buccolingual width, but it was less in the ridge preservation group. Thus, the use of G-graft with PRF and chorion membrane was highly effective in ridge preservation.

Key messages: Natural bovine bone mineral, along with PRF and chorion as a membrane, can be utilized effectively for ridge preservation in extracted tooth sockets due to periodontal disease.

## Introduction

The success of implant therapy depends on the prosthetic-driven position of the implant, bone to implant contact, an adequate number of ridge contours, and proper surgical procedure [1]. Tooth extraction and subsequent healing usually result in bony deformities, including reduced alveolar ridge height and reduced width with unfavorable ridge architecture for dental implant placement [2]. Ridge preservation is a surgical procedure carried out after extraction to prevent the collapse of the ridge and to preserve the ridge dimension, as usual, for implant site development [3]. For this purpose, allografts, alloplastics, and xenograft materials, along with autogenous bone, can be utilized [4-6].

The addition of platelet-rich fibrin (PRF) to the graft materials enhances wound healing and hemostasis, bone growth and maturation, and bone density, which impart better handling properties to the graft materials [7]. In vitro studies revealed that PRF induces various cell proliferation with the most potent induction effect on osteoblasts [8].

The chorion membrane acts as a barrier for scaffolds in tissue regeneration. It is rich in type I, IV, V, and VI collagens, proteoglycans, fibronectin, and laminin [9]. These allogenic membranes have antibacterial, antimicrobial, anti-inflammatory properties, very low immunogenicity, and are demonstrated to enhance gingival biotype [10]. Very limited scientific data are available regarding the use of PRF and the chorion membrane (Tissue Bank, Tata Memorial Hospital, Mumbai, India) for the preservation of ridges in a large sample size. Thus, the present study aims to compare the efficacy of G-graft, platelet-rich fibrin, and chorion membrane in post-extraction sockets (test group) with extraction sites alone (control group).

## Materials and methods

The study was a randomized case-control pilot design with approval from the institutional ethical committee. The study sample was obtained from the Out-Patient Department of Periodontics at G. Pulla Reddy Dental College and Hospital, Kurnool. A healthy subject with extraction sites should have neighboring teeth on both sides and extraction defect sounding (EDS) 3-4 were included. Patients with systemic diseases, aggressive periodontitis, pregnancy and lactating mothers, patients receiving chemotherapy and radiation therapy, current smokers or a history of smoking, and patients receiving long-term steroidal or antibiotic therapy were all excluded. Initially, 50 subjects were evaluated for inclusion criteria; among them, 12 patients were excluded from the study as they did not meet the study criteria and eight people were unwilling to participate.

Study design

A total of 30 subjects were included, in whom 60 sites were treated. The selected subjects were explained regarding the treatment. Written consent was obtained. The patient's detailed medical and dental history was recorded. The selected sites were randomly assigned utilizing a coin toss method into groups I (control groups) and II (test groups). All clinical measurements were preoperatively and postoperatively performed by single observers without the knowledge of treatment groups.

In the control group, the extraction sites were allowed to heal by a natural process. In the test group, socket preservation was done using hydroxyapatite with collagen (G-Graft), PRF, and chorion membrane.

All the clinical parameters were measured at baseline and at six months of follow-up: plaque index (Silness and Loe, 1964), gingival index (Loe and Silness index, 1963), and horizontal width were measured at baseline as the distance between the buccal and lingual plates (buccolingually) with a bone caliper. At six months, using the occlusal stent as a guide mark, a point 2 mm apical to the lingual and buccal bone plates at mid-portion mesiodistally for measurements. Vertical ridge height was also measured at baseline and six months. Measurements were obtained through markings made on the coronal portion of the stent at the mid-portion of the socket, on the buccal plate, and the lingual plate using endodontic reamers with a rubber stopper [11].

**Figure 1:**
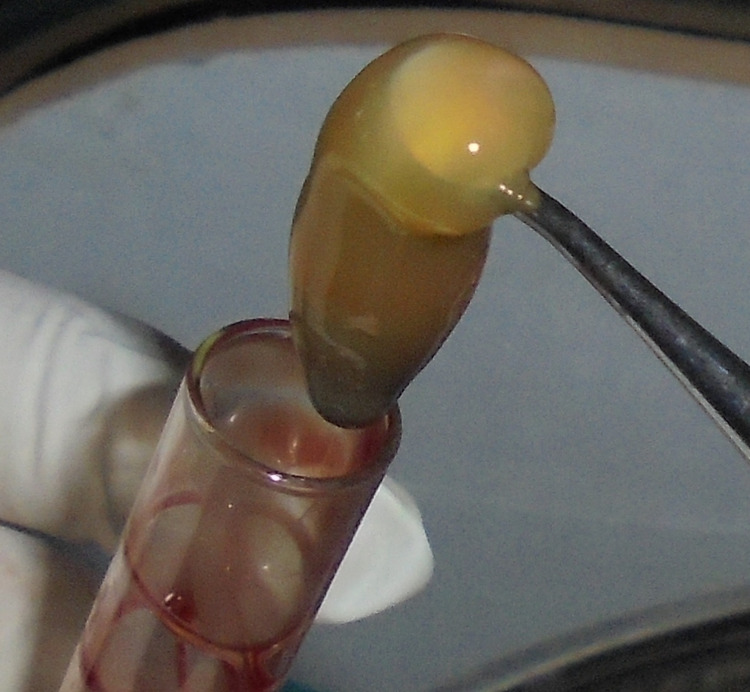
Platelet-rich fibrin obtained.

Immediately before surgery, about 10 ml of intravenous blood was obtained from the antecubital vein of the patients by venipuncture into two sterile vacutainer tubes without any anticoagulant. The vacutainer tubes are put in a centrifuge machine and spun at 3000 revolutions per minute for 10 minutes, resulting in the formation of an organized fibrin clot at the center of the vacutainer, just between the red corpuscles at the bottom and acellular plasma (platelet-poor plasma (PPP)) at the top. PRF clump, hence formed, was isolated utilizing sterile tweezers and scissors and used with graft material (Figure 1) [12].

**Figure 2:**
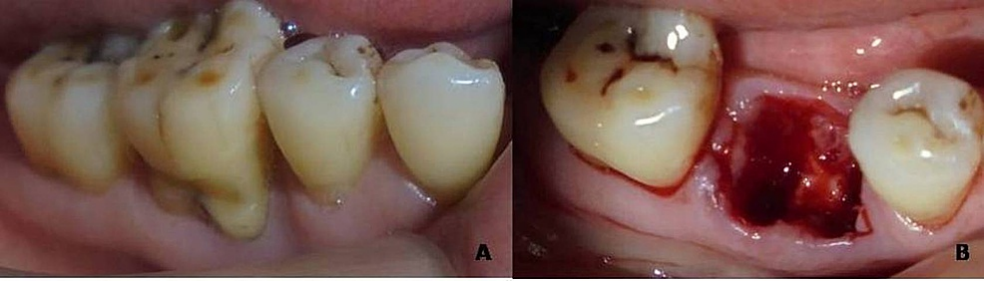
(a) Tooth indicated for extraction and (b) after atraumatic extraction.

After administering local anesthesia, a crevicular incision was made around the tooth to be extracted so that it should extend one tooth mesially and one tooth distally with no more than 15 blades. A mucoperiosteal flap was reflected using a periosteal elevator. Periodontal fibers around the tooth were resected using periotomes, and the tooth was then extracted atraumatically (Figure 2a, 2b). The socket was thoroughly debrided with Gracey curettes and bone curettes. At this point, baseline measurements, i.e., horizontal width and vertical height of the alveolar ridge with bone calipers and endodontic reamers using an acrylic stent, respectively, were measured. Papillae were preserved during the treatment procedures so that there was a close adaptation of the flap to the crestal alveolar bone. No effort was made to cover the extracted tooth socket or chorion layer.

**Figure 3:**
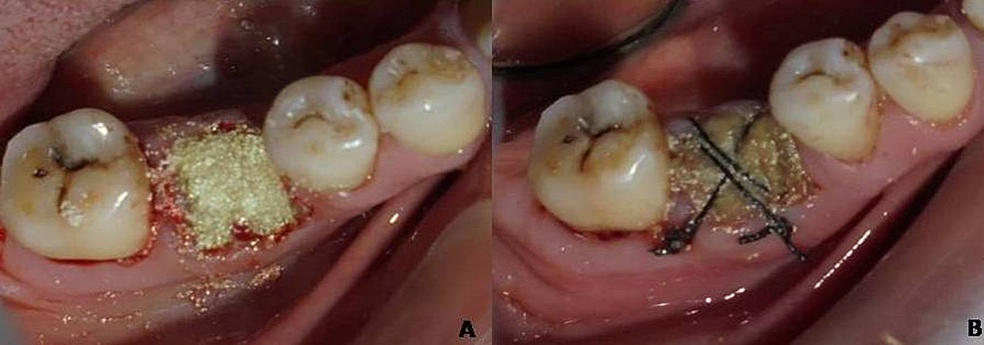
(a) Graft mixed with platelet-rich fibrin placed in the extraction socket and (b) chorion membrane placement and secured with sutures.

In group I, after debridement of the socket, no additional treatment was performed. In group II, after debridement of the socket, hydroxyapatite and collagen mixed with PRF were inserted into the socket and covered by a chorion membrane, and the membrane was secured with a non-absorbable suture material (Figure 3a, 3b).

**Figure 4:**
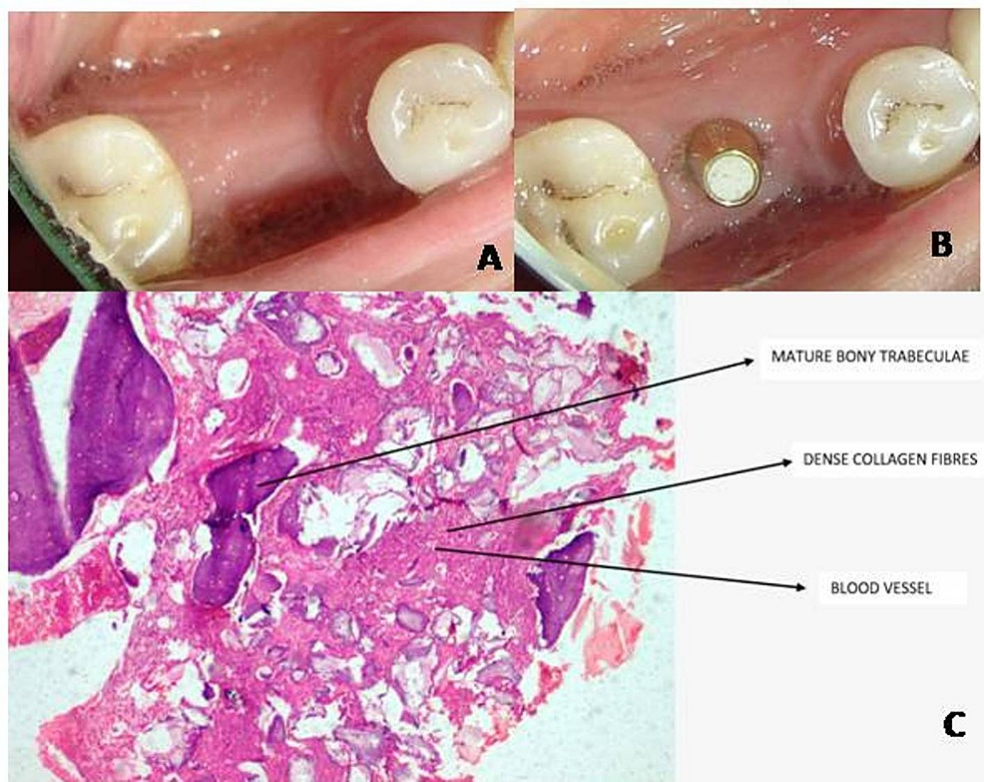
(a) Six months postoperative, (b) implant placements after six months, (c) six months histological image at x10 for test group and arrows represents mature bony trabeculae, dense collagen fibers, blood vessels, and remnants of bone graft.

Standard post-surgical instructions were given, and they prescribed Amoxicillin 500 mg three times a day (TID) for five days and an analgesic drug (Ibuprofen, 400 mg) twice daily for three days to reduce postoperative pain and discomfort. Suture removal was done after 10 days, wound healing was evaluated, and oral hygiene instructions were reinforced. Again, all the clinical parameters were assessed six months postoperatively, followed by implant placement (Figure 4a). During implant placement, a trephine core biopsy was procured from the ridge preservation site and sent for histologic analysis, which showed mature bony trabeculae within the fibrous tissue. The fibrous tissue contains dense collagen fibers and blood capillaries. Foci of structureless, eosinophilic deposits are evident, possibly remnants of bone graft (Figure 4b).

All the data recorded were subjected to statistical analysis. The final outcomes were averaged (mean ± S.D.) for every parameter and are presented in tables. Intragroup comparison of plaque and gingival indices and other clinical parameters among the two groups was made using the paired t-test. Intergroup comparison of parameters was analyzed using an unpaired t-test. In all the above clinical parameters, a P-value < 0.05 was considered to be statistically significant.

## Results

Initially, 50 subjects were enlisted, and 30 subjects completed the course uneventfully. In group I, 45% of subjects were male and 55% were female, whereas in group II, 43% were male and 57% were female. The mean age of the subjects in group I was 34.27 ± 7.35 years when compared to 35.46 ± 7.36 years for subjects in group II. Statistically, the two categories were matched for age and gender. Both groups showed a significant reduction in plaque and gingival indices from baseline to six months (Tables 1-2). There was no statistically significant difference between the two groups (Table 3).

**Table 1:** The comparison of all the parameters from baseline to six months within the control group using the Student's t-test. P < 0.05: considered statistically significant. SD: standard deviation; PI: plaque index; GI: gingival index; BLW: buccolingual width; BBP Ht.: buccal bone plate height; LBP Ht.: lingual bone plate height; P: probability value; t: Student's t-test value.

Group I	Baseline		Six months		t-value	P-value
	Mean	SD	Mean	SD		
PI	0.9282	0.2284	0.6727	0.1406	6.5991	<0.001
GI	1.9147	0.2670	1.0660	0.2912	16.2700	<0.001
BLW	8.5000	1.3496	5.5667	1.0668	18.2363	<0.001
BBP Ht.	15.5667	1.5683	17.6667	1.4100	13.4755	<0.001
LBP Ht.	15.3333	1.8581	17.2667	1.9167	20.1494	<0.001
Mesial socket	20.8667	2.0569	16.5000	2.4713	11.9091	<0.001
Distal socket	21.3333	1.9791	16.8333	2.3120	12.4353	<0.001

**Table 2:** The comparison of all the parameters from baseline to six months within the test group using Student’s t-test. *Not significant; P < 0.05: considered statistically significant. SD: standard deviation; PI: plaque index; GI: gingival index; BLW: buccolingual width; BBP Ht.: buccal bone plate height; LBP Ht.: lingual bone plate height; P: probability value; t: Student's t-test value.

Group II	Baseline		Six months		t-value	P-value
	Mean	SD	Mean	SD		
PI	0.9562	0.2077	0.7207	0.0700	5.2174	0.0001
GI	1.9737	0.0793	0.9453	0.1916	23.5655	<0.001
BLW	8.6667	1.4351	6.8667	1.2602	18.9237	<0.001
BBP Ht.	16.5667	2.4118	17.1333	2.6150	1.9667	0.0694*
LBP Ht.	15.9667	2.7155	16.8333	2.8452	2.7921	0.0144
Mesial socket	22.7000	1.3066	14.7667	0.9796	38.4644	<0.001
Distal socket	22.4000	1.1832	14.5333	1.1872	47.6098	<0.001

**Table 3:** The comparison of all the parameters between two groups using unpaired t-test. *Not significant; P < 0.05: considered statistically significant. SD: standard deviation; PI: plaque index; GI: gingival index; BLW: buccolingual width; BBP Ht.: buccal bone plate height; LBP Ht.: lingual bone plate height; P: probability value; t: unpaired t-test value.

		Group I		Group II		t-value	P-value
		Mean	SD	Mean	SD		
PI	Baseline	0.9282	0.2284	0.9562	0.2077	0.3513	0.7280*
	6 months	0.6727	0.1406	0.7207	0.0700	1.1832	0.2467*
GI	Baseline	1.9147	0.2670	1.9737	0.0793	0.8203	0.4190*
	6 months	1.0660	0.2912	0.9453	0.1916	1.3407	0.1908*
BLW	Baseline	8.5000	1.3496	8.6667	1.4351	0.3277	0.7456*
	6 months	5.5667	1.0668	6.8667	1.2602	3.0494	0.0050
BBP Ht.	Baseline	15.5667	1.5683	16.5667	2.4118	1.3463	0.1890*
	6 months	17.6667	1.4100	17.1333	2.6150	0.6953	0.4926*
LBP Ht.	Baseline	15.3333	1.8581	15.9667	2.7155	0.7455	0.4622*
	6 months	17.2667	1.9167	16.8333	2.8452	0.4892	0.6285*
Mesial socket	Baseline	20.8667	2.0569	22.7000	1.3066	2.9138	0.0069
	6 months	16.5000	2.4713	14.7667	0.9796	2.5253	0.0175
Distal socket	Baseline	21.3333	1.9791	22.4000	1.1832	1.7917	0.0840*
	6 months	16.8333	2.3120	14.5333	1.1872	3.4274	0.0019

In group I, the mean baseline buccolingual width was 8.50 ± 1.34 mm, which reduced to 5.56 ± 1.06 mm at nine months, and this reduction was statistically significant (Table 1). Ridge preservation sites had a mean initial buccolingual width of 8.66 ± 1.43 mm that reduced to 6.86 ± 1.26 mm at nine months, and this reduction was statistically significant (Table 2). Intergroup comparison for the difference in the buccolingual width was statistically significant with a mean change in group I (3 ± 0.62 mm) and group II (1.8 ± 0.36 mm). Intergroup comparison for the amount of socket depth filled in group I for mesial and distal sockets, respectively, was 4.36 ± 1.42 mm; 4.5 ± 1.4 mm; in group II, it was 7.93 ± 0.79 mm; 7.86 ± 0.63 that was statistically significant (p < 0.05). No statistically significant difference between the two groups for buccal and lingual bone plate height was observed (P > 0.05; Table 3). All the comparative changes and statistical analysis between the two groups were enlisted in Table 4.

**Table 4:** The changes observed from baseline to six months between two groups using the unpaired t-test. P < 0.05: considered statistically significant. SD: standard deviation; BLW: buccolingual width; BBP Ht.: buccal bone plate height; LBP Ht.: lingual bone plate height; P: probability value; t: unpaired t-test value.

Changes observed from baseline to six months	Group I		Group II		t-value	P-value
	Mean	SD	Mean	SD		
BLW	3.00	0.60	1.80	0.35	6.39	<0.05
BBP Ht.	2.10	0.60	1.10	0.54	4.60	<0.05
LBP Ht.	1.93	0.37	0.86	1.20	3.28	<0.05
Mesial socket	4.36	1.42	7.93	0.79	−8.47	<0.05
Distal socket	4.50	1.40	7.86	0.63	−8.46	<0.05

## Discussion

Alveolar bone, being a structural bone, is predictably lost following tooth extraction. There is enough evidence to state that major changes in the ridge dimensions may occur within 12 months, accounting for about 50% of the loss, and two-thirds of this happening within the first three months. Ridge preservation follows the principles of guided regeneration and uses structural scaffolds. The procedure uses regenerative materials and aims to reduce the resorption of the ridge and improve bone formation within the tooth socket. In very few clinical situations, like a tooth with acute infection, it might be delayed by six to eight weeks [13]. From their observations, Lekovic et al. said that there might be a more significant reduction in ridge width than height, and bone loss of some degree was noted at all extraction sites [14,15]. 

In our study, both groups showed a remarkable reduction in plaque and gingival indices from baseline to six months; however, there was no statistically significant distinction between the two groups. Regular recall visits result in the good overall maintenance of oral hygiene and, if required, underwent supragingival scaling. No post-surgical complications like pain, swelling, or dry socket were observed during the treatment. An adequate flap elevation was made to get the required membrane coverage, but a complete flap approximation was not made. In their observations, Nam et al. [16] stated that membrane exposure does not have any influence on the outcomes of the treatment.

In our study, a reduction in the alveolar ridge width was noted in both the control and test groups. After six months following treatment, there was a significantly greater reduction in the horizontal width in group I than in group II. The horizontal changes observed in this study were in agreement with Iasella et al. [5]. The amount of socket depth fill was statistically significant in both the groups, with a maximum of 7.93 ± 0.798 mm in the test group and 4.5 ± 1.4 mm in the control group.

The graft material used in this study was bovine natural hydroxyapatite with collagen, which has several advantages over synthetic graft materials. It has a definitive regenerative potential, gets completely reabsorbed, and is replaced by native bone, enabling predictable bone regeneration [17] without enhancing any significant inflammatory process. Treated sites with this graft showed a gain in bone density at an early stage and also enhanced bone healing [18]. The particle size of this graft was also ideal for socket preservation, as mentioned in a review by Wang and Lang [19]. Therefore, G-Graft can be utilized for the preservation of the ridge, where the placement of an implant is planned at an early stage [18]. We added PRF to the graft material as it enhances the regenerative potential of the graft. Growth factors play an important role in cell proliferation, cell migration, differentiation, and extracellular matrix formation by signaling the molecules for tissue regeneration [19]. Shah et al. performed socket preservation with bone allograft and the human chorion membrane as a barrier in a 22-year-old male patient. They concluded that these materials proved to be effective in minimizing soft/hard tissue loss [20].

The strengths of the study included a predetermined type of extracted tooth socket; atraumatic extraction carried out using periotomes; the addition of PRF to the graft material; and a histologic examination carried out to determine the type of bone formed. The limitations of this study are that radiographic techniques were not considered. The results of the present study show that the ridge preservation approach (using hydroxyapatite with collagen, PRF, and chorion membrane) effectively reduces the hard tissue loss in the test group compared to the control group.

## Conclusions

From our study, we can conclude that natural bovine bone material, along with PRF and the chorion membrane, can be utilized effectively for ridge preservation procedures. Patients undergoing this treatment procedure had a good ridge form favorable for the placement of the fixed and removable prostheses, or even implant placement if necessary. Regardless of the reasons for socket preservation, clinicians must be aware that sufficient alveolar bone volume and favorable architecture of the alveolar ridge are essential to achieve ideal functional and esthetic prosthetic reconstruction following implant therapy. Future studies should focus on a large number of patients with long-term follow-up.
